# 0919. Effect of catecholamine immediately after blast lung injury caused by laser-induced shock wave in a mouse model

**DOI:** 10.1186/2197-425X-2-S1-O27

**Published:** 2014-09-26

**Authors:** H Miyawaki, D Saitoh, K Hagisawa, M Noguchi, S Satoh, M Kinoshita, H Miyazaki, Y Satoh, T Sakamoto

**Affiliations:** National Defense Medical College Hospital, Department of Traumatology & Critical Care Medicine, Tokorozawa, Japan; National Defense Medical College, Division of Traumatology, Research Institute, Tokorozawa, Japan; National Defense Medical College, Division of Physiology, Tokorozawa, Japan; National Defense Medical College, Division of Biomedical Information Sciences, Tokorozawa, Japan; National Defense Medical College, Department of Immunology and Microbiology, Tokorozawa, Japan; National Defense Medical College, Department of Anesthesiology, Tokorozawa, Japan

## Introduction

The physical damage inflicted by blast waves is called primary blast injury, and lungs are vulnerable to blast waves [1]. Blast lung injuries (BLI) can be extremely critical during the super-acute phase, and hypotension is supposed to be the main cause of death (1) , but its etiology has not been elucidated. Recent studies have demonstrated that hypotension is mediated by the absence of vasoconstriction [2]. However, research investigated the effectiveness of catecholamine for BLI during the super-acute phase was not identified.

## Objectives

The present study aimed to establish a small-animal model of severe BLI using laser-induced shock wave (LISW) and to evaluate the effect of catecholamine on the super-acute phase of severe BLI.

## Methods

The investigation comprised two parts. Study 1 assessed the validity of the BLI model using LISW as follows. Mice were randomly allocated to groups that received 1.2, 1.3 or 1.4 J/cm^2^ LISW. Survival rates, systolic blood pressure (sBP), heart rate (HR), and peripheral oxyhemoglobin saturation (SpO_2_) were monitored for up to 60 min thereafter and lung tissues were histopathologically analyzed. Study 2 evaluated the effects of catecholamines as follows. The mice were randomly assigned to groups that received 1.4 J/cm^2^ LISW followed by the immediate intraperitoneal administration of dobutamine, noradrenaline or normal saline. A sham group received no LISW or drugs. Survival rates were measured for 48 h. We also measured sBP, HR, and SpO_2_ before and 5 and 10 min after LISW, and left ventricular ejection fraction (EF) and systemic vascular resistance (SVR) before and 1 min after LISW.

## Results

(Study 1) The triad of BLI (hypotension, bradycardia, and hypoxemia) was evident immediately after LISW. The degree of the triad and the survival rates were aggravated with increasing doses of LISW. The histopathological findings were compatible with BLI.

(Study 2) The survival rate was highest in the group that received noradrenaline, with significantly elevated SVR and decreased EF after LISW.

## Conclusions

The LISW induced lung injury model seems to be useful as severe BLI in small animals without any large scaled equipment. The main cause of death during the super-acute phase of severe BLI might be hypotension due to the absence of peripheral vasoconstriction. The immediate administration of an α1-adrenergic receptor agonist such as noradrenaline right after exposure to blast waves might be an effective treatment during the super-acute phase of severe BLI.
